# Degradation kinetics of aromatic VOCs polluted wastewater by functional bacteria at laboratory scale

**DOI:** 10.1038/s41598-022-21356-4

**Published:** 2022-11-09

**Authors:** Ying Lv, Liangshi Wang, Xingyu Liu, Bowei Chen, Mingjiang Zhang

**Affiliations:** 1 National Engineering Research Center for Environment-Friendly Metallurgy in Producing Premium Non-Ferrous Metals, GRINM Group Co., Ltd., Beijing, 101407 China; 2 School of Metallurgical and Ecological Engineering, University of Science and Technology Beijing, Beijing, 100083 China; 3 GRINM Resources and Environment Tech. Co., Ltd., Beijing, 101407 China; 4 General Research Institute for Nonferrous Metals, Beijing, 100088 China; 5 College of Water Resources and Environment, China University of Geosciences, Beijing, 100083 China

**Keywords:** Biotechnology, Environmental sciences, Microbiology techniques

## Abstract

Reaction kinetics in biodegradation process is the basis and key of bioremediation technology, which can be used to predict the interaction between microorganisms and environmental states in the system. In this study, the kinetic model (Monod, Moser, Tessier and Cotonis) and kinetic parameters of aerobic biodegradation of functional bacteria in simulated wastewater polluted by aromatic volatile organic compounds (VOCs) were determined by shaking flask experiment. Monod, Moser, Tessier and Contois models were used to fit the experimental data and determine the kinetic parameters based on nonlinear regression analysis. Experimental results demonstrated that the removal rate of aromatic VOCs at 72 h was between 34.78 and 99.75% depending on the initial concentration of aromatic VOCs. The specific growth rate *μ* and degradation rate *q* increased with the increase of substrate concentration. The model of Monod, Moser and Tessier could be used to simulate microbial growth and substrate degradation in simulated aromatic VOCs polluted wastewater. Then the model and corresponding kinetic parameters were used to predict the limit concentration of biodegradation and provide theoretical support for the subsequent dynamic simulation and field engineering.

## Introduction

With the rapid development of the chemical industry and the extensive use of chemical products, the pollution situation of aromatic VOCs is becoming more and more serious^1,2^. Among them, benzene, toluene, ethylbenzene and *m*-xylene are considered to be the most common VOCs pollution in chemical contaminated sites, and recent domestic research reports also showed that chlorinated hydrocarbons including chlorobenzene, *p*-chlorotoluene and *p*-chlorotrifluorotoluene are also ubiquitous^3–6^. Among various treatment technologies for aromatic VOCs, microbial remediation technology has the advantages of simple operation process, high removal efficiency, low energy consumption, and no secondary pollution, becoming a new trend in the treatment of aromatic VOCs^7^. Researchers have found that numerous microorganisms in nature have the ability to degrade aromatic VOCs, but relevant studies predominantly focus on the screening of degrading strains, degradation characteristics and optimization of process conditions^8–10^. From the current research status, biodegradation research should not only focus on the study of microbial degradation performance, but also pay more attention to how to strengthen the process of microbial degradation, and the application of modeling method to promptly detect and evaluate microbial metabolism. Among them, reaction kinetics in the biodegradation process is the basis and key of microbial remediation technology^11^.

Kinetics of microbial reaction process is a quantitative description of the rate in the cell reaction process, including microbial growth rate, metabolite generation rate and substrate consumption rate^12,13^. It reflects the intrinsic kinetic characteristics of the reaction process at the cellular level, and is an important theoretical basis for optimizing the cell reaction process^14,15^. Consequently, the corresponding kinetic models mainly involve microbial growth, substrate degradation and product formation kinetic models. The construction of kinetic model can be used to predict the interaction between microorganisms and environmental conditions in the system, and can also optimize and simulate the degradation process, which is an indispensable and important tool in the field engineering of microbial degradation of organic pollution^16^.

Key variables should be selected according to the purpose when constructing a dynamic model. Therefore, the biodegradation process of pollutants is mainly studied in two aspects. Firstly, microorganisms use pollutants as carbon sources, and the consumption of pollutants caused by uptake leads to substrate degradation. The second is the growth of microbial cells themselves^17^. The corresponding kinetic model should also contain two aspects: microbial growth kinetics to describe the relationship between microbial growth and substrate concentration; the other is substrate degradation kinetics to describe the relationship between substrate degradation efficiency and concentration. By studying the degradation rate and reaction order, it is helpful to understand and analyze the degradation law and mechanism comprehensively. In addition, there are limit concentrations of microorganisms not only for common carbon sources, but also for other nutrients that can be used, such as some aromatic VOCs that can be biodegradable. Nevertheless, there is little research on limit concentrations of these organic compounds.

To fill these knowledge gaps, the model of Monod, Moser, Tessier and Cotonis were used in this study to simulate the microbial growth kinetics and aromatic VOCs degradation kinetics during the biodegradation process by functional bacteria. The specific objectives of this study were to investigate (1) the appropriate kinetics model by kinetic fitting comparation at laboratory scale; (2) the degradation ability and dominant potential of the constructed functional bacteria based on the specific kinetic parameters; and (3) provide theoretical basis and support for dynamic simulation and field engineering of aromatic VOCs treatment.

## Materials and methods

### Construction of functional bacteria

The functional bacteria used in this study included 5 strains, relating to *Rhodococcus qingshengii* LY-4 (with a reserve number of CCTCC M 2022520), *Pseudomonas* sp. LY-13 (with a reserve number of CCTCC M 2022521), *Aeromonas* sp. LY-45 (with a reserve number of CCTCC M 2022522), *Achromobacter* sp. LY-51 (with a reserve number of CCTCC M 2022523) and *Bacillus benzoevorans* (with a reserve number of CGMCC1.3665). The first four strains were screened from chemical contaminated sites, while *Bacillus benzoevorans* was purchased from the General Microbial Storage Center of Institute of Microbiology, Chinese Academy of Sciences.

Before the biodegradation experiment, the 5 strains were inoculated into LB medium at 30 °C and 150 r min^−1^ for rejuvenation. After the microorganisms grew to logarithmic stage, 1 mL of each bacterial solution was taken out and centrifuged at 6500 r min^−1^ for 10 min. After removing the supernatant solution, the thallus was washed twice with sterilized 0.9% normal saline to remove a small amount of medium on the surface of the thallus. Then the bacteria were dissolved in 1 mL 0.9% normal saline, and the functional bacteria was constructed after perfect mixing.

### Preparation of simulated wastewater containing aromatic VOCs

The aromatic VOCs used in the experiment include benzene, toluene, ethylbenzene, chlorobenzene, *m*-xylene, *p*-chlorotoluene and *p*-chlorotrifluorotoluene, with analytical purity. These aromatic VOCs were selected based on the current pollution characteristics of chemical contaminated sites, so that the research results have universal applicability. Considering that 7 types of aromatic VOCs are insoluble in water, ethanol was selected as the mixed solvent. Because *p*-chlorotoluene and *p*-chlorotrifluorotoluene have superior toxicity, the simulated wastewater prepared in this study had a lower mass concentration of these two organics. After preliminary tests, it was confirmed that 0.6 mL original mother liquor of aromatic VOCs contained 85.6 μL benzene, 86.7 μL toluene, 86.2 μL ethylbenzene, 68.2 μL chlorobenzene, 87.2 μL *m*-xylene, 58.4 μL *p*-chlorotoluene, 58.4 μL *p*-chlorotoluene, and 80.9 μL ethanol. That is, the mass concentration of the first 5 aromatic VOCs was 12,500 mg L^−1^, while the mass concentration of *p*-chlorotoluene and *p*-chlorotrifluorotoluene was 10,417 mg L^−1^. Eventually, the reaction system with known aromatic VOCs concentration is prepared by transferring different volumes of the original mother liquor into reaction system.

### Design of biodegradation experiments

Fill a series of 20 mL headspace bottles with 9 mL sterilized specific medium (121 °C for 30 min), then inoculate 1 mL of the functional bacteria with a pipette (the total reaction system was 10 mL). After that, the original mother liquor of aromatic VOCs was transferred into the headspace bottles with a volume of 0.3 μL, 0.6 μL, 0.9 μL, 1.2 μL, 1.8 μL, 2.4 μL and 3.0 μL (total aromatic VOCs concentration was 25, 50, 75, 100, 150, 200 and 250 mg L^−1^), respectively. *S*_*0*_ and *X*_*0*_ were determined immediately after the microorganisms was inoculated to the reaction system, and the samples were fixed on a flip oscillator and cultured for 72 h, under the condition of 30 °C and 30 r min^−1^ (rotational speed). Each group of experiments was set up in 6 parallel groups, among which 3 groups were used to test the mass concentration of aromatic VOCs (*S*), and the other 3 groups were used to measure biomass (*X*) after centrifugation and washing treatment. The biomass concentration *X* (mg L^−1^) was determined by transferring the solid phase of the sample after centrifugation to aluminum foil with constant weight and drying it in an oven at 105 °C for 8 h, then calculating the weight difference. The concentration of aromatic VOCs was determined by gas chromatography with headspace injection.

The components of the specific medium were as follows: Na_2_HPO_4_·2H_2_O 7 g, KH_2_PO_4_ 2 g, MgSO_4_·7H_2_O 0.2 g, glycerin 0.1 g, corn pulp powder 0.5 g, 1 mL mother liquor of trace elements, 1 L distilled water. The composition and concentration of mother liquor of trace elements were as follows: Ca(NO_3_)_2_·4H_2_O 600 mg L^−1^, MnSO_4_·4H_2_O 20 mg L^−1^, CuSO_4_·5H_2_O 40 mg L^−1^, H_3_BO_3_ 3 mg L^−1^, FeSO_4_·7H_2_O 200 mg L^−1^, NaMoO_4_·2H_2_O 4 mg L^−1^, ZnSO_4_·7H_2_O 20 mg L^−1^. 1 mol/L NaOH was used to adjust the pH value to 7.0, and then the specific medium sterilized at 121 °C for 30 min before use.

### Kinetic method

#### Microbial growth kinetic model

In the study of microbial growth kinetics, the change rates of biomass and substrate concentration can be calculated by Eqs. (1) and (2):

1 $$\frac{dX}{dt}={r}_{1}$$

2 $$\frac{dS}{dt}={-r}_{2}$$

And the specific growth rate can be calculated based on the change of biomass concentration in the kinetic experiment as shown in the following equation:

3 $$\mu =\frac{dX}{Xdt}=\frac{({X}_{e}-{X}_{0})}{{X}_{0}\Delta t}$$

Yield coefficient *Y*_*X/S*_ is defined as follows:

4 $${Y}_{X/S}=\frac{{X}_{e}-{X}_{0}}{{S}_{0}-{S}_{e}}=\frac{\mu }{q}$$

where *X* is biomass concentration, *t* represents experiment time (h), *X*_0_ indicates initial biomass concentration (mg L^−1^), *X*_*e*_ indicates final biomass concentration (mg L^−1^), *S*_*0*_ is initial substrate concentration (mg L^−1^), *S*_*e*_ is final substrate concentration (mg L^−1^), *μ* represents specific growth rate (h^−1^), *q* represents specific degradation rate (h^−1^), *Y*_*X/S*_ is yield coefficient, which is dimensionless.

There is a certain quantitative relationship between substrate concentration *S* and *μ*. These mathematical relationships can be expressed by microbial growth kinetic models including Monod, Moser, Tessier and Contois. Monod model can be described as Eq. (5):

5 $$\mu =\frac{{\mu }_{max}S}{{K}_{S}+S}$$

Moser, Tessier and Contois model can be described as Eqs. (6)–(8):

6 $$\mu =\frac{{\mu }_{max}{S}^{n}}{{K}_{MS}+{S}^{n}}$$

7 $$\mu ={\mu }_{max}(1-{e}^{-\frac{S}{{K}_{T}}})$$

8 $$\mu =\frac{{\mu }_{max}S}{{K}_{C}X+S}$$

where *µ*_*max*_ represents the maximum specific growth rate of microorganisms, t^−1^; *K* is the half saturation coefficient, mg L^−1^; *S* refers to the substrate concentration, mg L^−1^; *X* denotes biomass concentration, mg L^−1^; *n* is the exponential parameter of Moser model.

In the microbial treatment of wastewater, the death and decay of microorganisms are commonly caused by pH fluctuation, higher shear flow rate, lower substrate concentration and toxicity of wastewater. When cell decay is considered, the microbial growth model can be changed into:

9 $$\mu *=\mu -{K}_{d}$$

where μ* is the net specific growth rate of biomass. Cell decay actually occurs at all stages of the biodegradation process, although in some studies the decay coefficient is close to zero and thus negligible.

#### Substrate degradation kinetic model

Monod, Moser, Tessier and Contois models have also been used to describe the mathematical relationship between *S* and *q* in many studies on the kinetics of microbial treatment of wastewater. According to *Y*_*X/S*_ and *μ* calculated from the experimental results, the specific degradation rate *q* can be calculated according to Eq. (10):

10 $$q=\frac{\mu }{{Y}_{X/S}}$$

Based on this, the deformation of Eqs. (5)–(8) is:

11 $$q=\frac{{q}_{max}S}{{{K}_{S}}^{^{\prime}}+S}$$

12 $$q=\frac{{q}_{max}{S}^{n{^{\prime}}}}{{{K}_{MS}}^{^{\prime}}+{S}^{n{^{\prime}}}}$$

13 $$q={q}_{max}(1-{e}^{-\frac{S}{{K}_{T}{^{\prime}}}})$$

14 $$q=\frac{{q}_{max}S}{{{K}_{C}}^{^{\prime}}X+S}$$

where *q*_*max*_ is the maximum specific degradation rate (h^−1^); *K′* refers to the half-rate constant of substrate degradation (mg L^−1^);* n*′ represents the exponential parameter of Moser model, which is dimensionless.

#### Theoretical yield coefficient, net yield coefficient and kinetic limit concentration

Yield coefficient refers to the increase in microbial biomass synthesized by metabolizing substrate per unit mass during anabolism, which is a dimensionless constant for a specific reaction system^18^. Yield coefficient does not include the biomass reduced by microbial endogenic respiration, but to a certain extent, it can qualitatively reflect the growth rate and energy required by microbial growth. Based on the model and kinetic parameters of microbial growth kinetics and substrate degradation kinetics obtained in the study, theoretical yield coefficient *Y*_*T*_ and net yield coefficient *Y*_*N*_ of the entire degradation system can be calculated:

15 $${Y}_{T}=\frac{{\mu }_{max}}{{q}_{max}}$$

16 $${Y}_{N}=\frac{{\mu }_{max}*}{{q}_{max}}=\frac{{\mu }_{max}-{K}_{d}}{{q}_{max}}$$

When the substrate concentration is lower than a specific value in the degradation process, microorganisms cannot obtain enough energy from the substrate to maintain normal metabolic activities. The specific concentration of substrate is regarded as the limit concentration of biodegradation (named as *S*_*min*_, mg L^−1^)^19^. Based on microbial growth kinetic model, *S*_*min*_ can be measured by setting the growth rate to zero, at which point *S*_*min*_ is also known as the kinetic limit concentration.

#### Analytical method

The kinetic model was fitted with experimental data by nonlinear regression analysis. Parameter estimation is based on nonlinear least-square criterion. The fitting results were tested statistically, and the regression coefficient *R*^2^ and *F* test values were used to evaluate the goodness of fit of the model.

## Results and discussion

### Determination results of kinetic parameters of degradation

In this study, the initial concentration of aromatic VOCs was set at 25–250 mg L^−1^, which was subject to the actual measured values after the inoculation of functional bacteria. The ratio growth rate of biomass (*μ*) was calculated according to Eq. (3), and yield coefficient (*Y*_*X/S*_) and ratio degradation rate (*q*) under different initial concentration were calculated according to Eqs. (4) and (10). The results of removal rate, *μ* and *q* after 72 h reaction are shown in Table 1. It can be seen that when the initial concentration is in the range of 26.137 mg L^−1^ to 101.558 mg L^−1^, the removal rate of aromatic VOCs was higher, which was above 75%. This may be due to the insignificant inhibitory effect of low concentration of aromatic VOCs on microbial growth, just as our previous study found that the degradation system with low concentration of aromatic VOCs had higher microbial activity. However, when the initial concentration was further increased, the removal rate decreased significantly. This might be due to the inhibitory effect of toxic aromatic VOCs on the growth of functional microorganisms. In addition, *μ* and *q* increased with the increase of substrate concentration. Especially, compared with the group with aromatic VOCs concentration of 26.137 mg L^−1^, *μ* increased by an order of magnitude in the group with aromatic VOCs concentration of 199.654 mg L^−1^.

**Table 1:** Changes of *μ*, *q* and removal rate of aromatic VOCs under different initial concentration.

Group	Substrate (mg L^−1^)		Biomass (mg L^−1^)		Removal rate (%)	*μ*/h^−1^	*q*/h^−1^
	*S*_*0*_	*S*_*e*_	*X*_*0*_	*X*_*e*_			
1	251.469	164.008	156.0	256.8	34.78	0.00897	0.00779
2	199.654	114.981	156.0	251.6	42.41	0.00851	0.00754
3	152.347	71.177	156.0	239.1	53.28	0.00740	0.00723
4	101.558	23.722	156.0	213.4	76.64	0.00511	0.00693
5	73.968	11.635	156.0	206.3	84.27	0.00448	0.00555
6	50.482	0.126	156.0	196.4	99.75	0.00360	0.00448
7	26.137	0.358	156.0	165.6	98.63	0.00085	0.00230

### Fitting of microbial growth kinetic model

**Figure 1:**
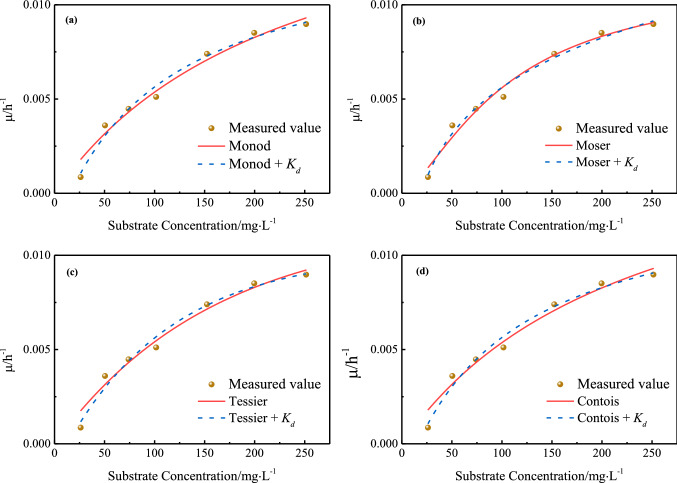
Nonlinear regression of microbial growth with and without cell decay using (**a**) Monod model, (**b**) Moser model, (**c**) Tessier model and (**d**) Contois model.

Based on the experimental data in Table 1, classical Monod, Moser, Tessier and Cotonis models were used in this study to simulate the functional relationship between *μ* and *S*. Monod model is the most widely used mathematical model for microbial treatment of wastewater^20^. Tessier model and Monod model have similar fitting curves^21^. Moser model introduces the parameter *n* into Monod model to provide a better fit for the data^22^. One of the purposes of this study was to find out the best model by comparative goodness of fit. Considering the inhibitory effect of toxic aromatic VOCs on microbial growth, the cell decay coefficient *K*_*d*_ was introduced in the fitting process. To ensure the fitting effect, the initial value of *μ*_*max*_ was set as 0.02 h^−1^, and the initial value of *K*_*d*_ was set as 0.01 h^−1^. Figure 1 illustrates the results of nonlinear regression analysis of microbial growth with and without cell decay.

It can be preliminarily judged from Fig. 1 that the simulation effects of the 4 models were all ideal, as the difference between the theoretical value and the measured value was not significant. Table 2 displays the corresponding values of the kinetic parameters based on each model and presents the results of statistical tests. Larger *R*^2^ (close to 1) and *F* values indicate better fitting degree of the model^16^. It can be found that the model taking cell decay into account showed a better fit to the data than the microbial growth model without *K*_*d*_. Therefore, cell decay due to the high toxicity of simulated wastewater should not be ignored in this study. At the same time, statistical test results demonstrated that all the models considering the cell decay showed modest differences in the fitting degree, indicating that they could be used to simulate the microbial growth of functional bacteria in theory. In particular, Contois model is commonly used to simulate microbial growth at high biomass densities^23^. However, in this study, biomass was maintained at a low level due to the toxic effects of aromatic VOCs (with a highest concentration of 256.8 mg L^−1^), and the *K*_*C*_ value provided by Contois model was found to be relatively low in the experimental results. Therefore, Contois model was not suitable for this study. In addition, Monod, Moser, and Tessier models, which consider the cell decay, could be used to describe the growth kinetics of the functional bacteria because of their good fit to the experimental data and reasonable kinetic parameters. In particular, the Monod model provided the best fit, with the largest *R*^2^ value of 0.9777. In fact, these three models are based on different assumptions, so these models can be applied according to different real-world situations.

**Table 2:** Kinetic parameters and statistic results from the nonlinear regression analysis of *μ* versus *S.*

Kinetic model		Kinetic parameter	Value	(*R*^2^)	*F*	*Prob* > *F*
Monod	$\mu =\frac{{\mu }_{max}S}{{K}_{S}+S}$	*μ*_*max*_*/*h^−1^	0.0181	0.9655	352.72	4.25 × 10^–5^
		*K*_*S*_/mg L^−1^	236.51			
Monod + *K*_*d*_	$\mu =\frac{{\mu }_{max}S}{{K}_{S}+S}-{K}_{d}$	*μ*_*max*_*/*h^−1^	0.0158	0.9777	468.41	1.81 × 10^–6^
		*K*_*S*_/mg L^−1^	103.46			
		*K*_*d*_/ h^−1^	0.0021			
Moser	$\mu =\frac{{\mu }_{max}{S}^{n}}{{K}_{MS}+{S}^{n}}$	*μ*_*max*_*/*h^−1^	0.0116	0.9706	255.11	3.14 × 10^–4^
		*K*_*MS*_/mg L^−1^	863.94			
		*n*	1.45			
Moser + *K*_*d*_	$\mu =\frac{{\mu }_{max}{S}^{n}}{{K}_{MS}+{S}^{n}}-{K}_{d}$	*μ*_*max*_*/*h^−1^	0.1368	0.9744	305.69	1.14 × 10^–5^
		*K*_*MS*_/mg L^−1^	7.46			
		*K*_*d*_/ h^−1^	0.0241			
		*n*	0.16			
Tessier	$\mu ={\mu }_{max}(1-{e}^{-\frac{S}{{K}_{T}}})$	*μ*_*max*_*/*h^−1^	0.0116	0.9686	297.78	3.36 × 10^–4^
		*K*_*T*_/mg L^−1^	159.87			
Tessier + *K*_*d*_	$\mu ={\mu }_{max}(1-{e}^{-\frac{S}{{K}_{T}}})-{K}_{d}$	*μ*_*max*_*/*h^−1^	0.0114	0.9752	420.54	2.24 × 10^–6^
		*K*_*T*_/mg L^−1^	107.59			
		*K*_*d*_/ h^−1^	0.0013			
Contois	$\mu =\frac{{\mu }_{max}S}{{K}_{C}X+S}$	*μ*_*max*_*/*h^−1^	0.0181	0.9655	452.72	4.25 × 10^–4^
		*K*_*C*_/mg L^−1^	1.26			
Contois + *K*_*d*_	$\mu =\frac{{\mu }_{max}S}{{K}_{C}X+S}-{K}_{d}$	*μ*_*max*_*/*h^−1^	0.0158	0.9777	468.41	1.81 × 10^–6^
		*K*_*C*_/mg L^−1^	0.66			
		*K*_*d*_/ h^−1^	0.0021			

*μ*_*max*_ represents the maximum potential of substrate utilization. The smaller the *K* value is, the stronger the inhibition effect will be when the substrate concentration is lower^23^. The growth kinetics parameters obtained in this study were compared with the data obtained in previous studies. For example, when functional bacteria were used to conduct aerobic biodegradation of benzene, toluene and ethylbenzene, Ma found that the maximum growth rate *μ*_*max*_ was 0.228 d^−1^, 0.192 d^−1^, 0.168 d^−1^, *K* value was 176.5, 125.4 and 105.4 mg L^−1^, respectively^24^; Sun found that when benzene, toluene, ethylbenzene and xylene were simultaneously present in a reaction system, competitive inhibition would occur, and the rate constants of benzene, toluene, ethylbenzene and xylene were 0.0286 h^−1^, 0.0681 h^−1^, 0.0631 h^−1^ and 0.0370 h^−1^, respectively^25^. In this study, taking the Monod model for example, the *μ*_*max*_ was 0.4344 d^−1^ (0.0181 h^−1^ × 24) and *K*_*S*_ value was 236.51 mg L^−1^. Considering that the substrates involved highly toxic chlorobenzene, *p*-chlorotoluene and *p*-chlorotrifluorotoluene, and the competitive inhibition caused by multiple components, it can be inferred that the constructed functional bacteria had significant degradation potential and tolerance to aromatic VOCs.

### Fitting of substrate degradation kinetic model

**Figure 2:**
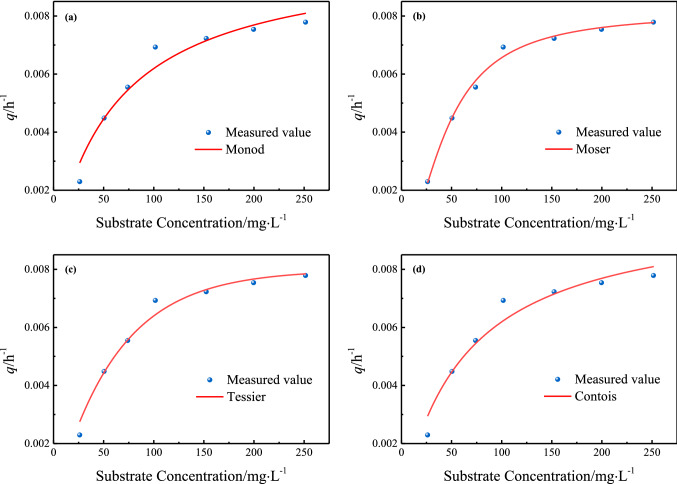
Nonlinear regression of substrate degradation using (**a**) Monod model, (**b**) Moser model, (**c**) Tessier model and (**d**) Contois model.

Equations (11)–(14) were used to fit the data of *q* and *S*. Figure 2 shows the results of nonlinear regression analysis of substrate degradation. The values of kinetic parameters obtained and the results of statistical tests are listed in Table 3. It can be seen that substrate degradation could be described by a semi-empirical microbial growth kinetics model, because the *Prob* > *F* values of all models used for fitting were less than 0.01 and *R*^2^ values were above 0.90, both of which indicated that the experimental results and curve fitting were ideal. None of the Monod, Moser, Tessier and Contois models had a significant advantage in statistical tests. In particular, Moser model showed the best fit, and the kinetic parameters of Monod, Moser and Tessier models were all within a reasonable range. *K*_*C*_^′^, however, obtained based on Contois model, was only 0.3421, which was far smaller than other models. Considering the fitting results of microbial growth, Monod, Tessier and Moser models could be used to describe substrate degradation and biomass growth simultaneously, so it could be concluded that these three models provided a complete set of kinetic parameters. Particularly, when Moser model was used to simulate microbial growth and substrate degradation respectively, different values of *n* were obtained, proving that *S* played different weights in the two models.

**Table 3:** Kinetic parameters and statistic results from the nonlinear regression analysis of *q* versus *S*.

Kinetic model		Kinetic parameter	Value	*R*^2^	*F*	*Prob* > *F*
Monod	$q=\frac{{q}_{max}S}{{{K}_{S}}^{^{\prime}}+S}$	*q*_*max*_*/*h^−1^	0.0102	0.9480	51.35	1.72 × 10^–6^
		*K*_*S*_^′^/mg L^−1^	63.96			
Moser	$q=\frac{{q}_{max}{S}^{n}}{{{K}_{MS}}^{^{\prime}}+{S}^{n}}$	*q*_*max*_*/*h^−1^	0.0081	0.9893	77.62	8.88 × 10^–7^
		*K*_*MS*_^′^/mg L^−1^	872.54			
		*n*^′^	1.78			
Tessier	$q={q}_{max}(1-{e}^{-\frac{S}{{K}_{T}{^{\prime}}}})$	*q*_*max*_*/*h^−1^	0.0080	0.9763	43.15	2.43 × 10^–7^
		*K*_*T*_^′^/mg L^−1^	61.8558			
Contois	$q=\frac{{q}_{max}S}{{{K}_{C}}^{^{\prime}}X+S}$	*q*_*max*_*/*h^−1^	0.0102	0.9480	51.35	1.72 × 10^–6^
		*K*_*C*_^′^/mg L^−1^	0.3421			

Monod is the most widely used model in substrate degradation kinetics. The kinetic parameters of this study were compared with the reported results of biodegradation of aromatic VOCs polluted wastewater. *q*_*max*_ in this study was 0.0102 h^−1^, lower than the kinetic results of toluene degradation studied by Sun^26^ and Men^27^ (with *q*_*max*_ of 0.0479 h^−1^ and 0.0385 h^−1^, respectively). As is well-known, *q*_*max*_ reflects the degradation potential of the bacteria. Therefore, the functional bacteria used in this study showed a slightly lower degradation rate for aromatic VOCs, which might be related to the toxicity and complexity of organic compounds in the culture system. *K*_*S*_^′^ in this study was 63.96 mg L^−1^, lower than the results of Chen et al.^28^ (806.1 mg L^−1^). The lower *K*_*S*_^′^ value indicated that the functional bacteria had a higher affinity for wastewater polluted by aromatic VOCs.

### Calculation of ***Y***_***T***_, ***Y***_***N***_ and ***S***_***min***_

Based on the kinetic parameters obtained in the nonlinear regression analysis, the theoretical and net yield coefficients were calculated by Eqs. (15) and (16), and the specific results are shown in Table 4. When Monod model was used to simulate the degradation kinetics of wastewater polluted by aromatic VOCs, *Y*_*T*_ was 1.775 and *Y*_*N*_ was 1.343. The results obtained by Cotois model were the same. In addition, the lowest *Y*_*T*_ and *Y*_*N*_ values were calculated based on Tessier model, were 1.450 and 1.263, respectively. In Moser model, the yield coefficient could not be directly calculated due to the difference of *n* values.

**Table 4:** Calculation results of *Y*_*T*_, *Y*_*N*_ and *S*_*min*_ based on different models.

Kinetic model	Monod	Moser	Tessier	Contois
*Y*_*T*_	1.549	–*	1.425	1.549
*Y*_*N*_	1.343	–	1.263	1.343

*The yield coefficient cannot be calculated directly because the value of parameter *n* is different.

When the substrate concentration decreases to a certain value, the energy generated by degradation cannot meet the needs of bacterial growth, and the growth and death of bacteria reach a dynamic balance^19^. At this time, the kinetic limit concentration *S*_*min*_ (the minimum concentration of biodegradation) can be calculated by setting the net ratio growth rate in Eq. (9) to 0. *S*_*min*_ reflects the lowest concentration of organic pollutants that can be achieved during biodegradation. In this study, when using Monod model, the following equation was assumed:

$$\mu =\frac{0.0158{S}_{min}}{103.46+{S}_{min}}-0.0021=0$$

*S*_*min*_ was calculated to be 15.86 mg L^−1^. When using Moser and Tessier model, respectively, the equation for *μ* was:

$$\mu =\frac{0.1368{{S}_{min}}^{0.16}}{7.46+{{S}_{min}}^{0.16}}-0.0241=0$$

$$\mu =0.0114(1-{e}^{-\frac{{S}_{min}}{107.59}})-0.0013=0$$

*S*_*min*_ were calculated to be 18.53 mg L^−1^ and 13.03 mg L^−1^, respectively. It can be seen that there were differences in the calculated concentrations among different models, but the differences were not significant, also confirming that Monod, Moser and Tessier models could provide a complete set of kinetic parameters for the biodegradation of aromatic VOCs. The determination of limit concentration is of great significance and practical value for the development of microbial purification technology, the evaluation of the degree of biochemical reaction, the development of biodegradation potential, the search for ways to enhance the efficiency of microbial treatment and the remediation of polluted environment.

## Conclusion

In this study, the degradation of aromatic VOCs at different initial concentrations was explored from the perspective of kinetics, and mathematical models were constructed to describe the treatment performance of the microbial system, so as to provide appropriate parameters for the treatment design of the polluted wastewater containing aromatic VOCs. Through the determination of kinetic parameters during the degradation process, it was found that when the initial concentration was in the range of 26.137–101.558 mg L^−1^, the removal rate of aromatic VOCs was higher, all above 75%. However, when the initial concentration was further increased, the removal rate showed an obvious trend of decrease. At the same time, *μ* and *q* increased with the increase of substrate concentration, and reached the maximum when the initial concentration of aromatic VOCs was 251.469 mg L^−1^. Nonlinear regression analysis of experimental results using four kinetic models revealed that Monod, Moser and Tessier models could be used to simulate microbial growth and substrate degradation in aromatic VOCs polluted wastewater. In particular, there was cell decay when simulating microbial growth, and the introduction of *K*_*d*_ could provide a better fit. And the values of *μ*_*max*_ and *K*_*S*_ showed some advantages compared with the existing research. Therefore, it could be inferred that the constructed functional bacteria had significant degradation potential and tolerance to aromatic VOCs. For the fitting of substrate degradation kinetic model, Moser model showed the best fit, and the kinetic parameters of Monod, Moser and Tessier models were all within a reasonable range. On the whole, Monod, Tessier and Moser models could provide a complete set of kinetic parameters for the biodegradation of aromatic VOCs, which could better describe the microbial growth and substrate degradation process. In addition, the model and kinetic parameters obtained by nonlinear regression analysis and statistical tests had been used to predict the biodegradation limit concentration (*S*_*min*_) of aromatic VOCs polluted wastewater. The *S*_*min*_ values obtained by Monod model, Moser model and Tessier model were 115.86 mg L^−1^, 18.53 mg L^−1^ and 13.03 mg L^−1^, respectively. These outcomes can provide theoretical support for future dynamic simulation and field engineering.

## Data Availability

The datasets used and/or analyzed during the current study are available from the corresponding author on reasonable request.
